# A study of head and neck cancer treatment and survival among indigenous and non-indigenous people in Queensland, Australia, 1998 to 2004

**DOI:** 10.1186/1471-2407-11-460

**Published:** 2011-10-25

**Authors:** Suzanne P Moore, Adèle C Green, Gail Garvey, Michael D Coory, Patricia C Valery

**Affiliations:** 1 Cancer and Population Studies, Queensland Institute of Medical Research, Herston Rd, Brisbane, 4060, Australia; 2 The Australian Centre for International and Tropical Health, University of Queensland, Herston Rd, Brisbane, 4006, Australia; 3 School of Population Health, University of Queensland, Herston Rd, Brisbane, 4006, Australia; 4 Murdoch Children's Research Institute, Flemington Rd, Melbourne, 3052, Australia; 5 Manchester Academic Health Science Centre, University of Manchester, Brunswick St, Manchester, M13 9PL, UK

## Abstract

**Background:**

Overall, Indigenous Australians with cancer are diagnosed with more advanced disease, receive less cancer treatment and have poorer cancer survival than non-Indigenous Australians. The prognosis for Indigenous people with specific cancers varies however, and their prognosis for cancers of the head and neck is largely unknown. We therefore have compared clinical characteristics, treatment and survival between Indigenous and non-Indigenous people diagnosed with head and neck cancer in Queensland, Australia.

**Methods:**

Rates were based on a cohort of Indigenous people (n = 67), treated in public hospitals between 1998 and 2004 and frequency-matched on age and location to non-Indigenous cases (n = 62) also treated in the public health system. Data were obtained from hospital records and the National Death Index. We used Pearson's Chi-squared analysis to compare categorical data (proportions) and Cox proportional hazard models to assess survival differences.

**Results:**

There were no significant differences in socioeconomic status, stage at diagnosis or number and severity of comorbidities between Indigenous and non-Indigenous patients, although Indigenous patients were more likely to have diabetes. Indigenous people were significantly less likely to receive any cancer treatment (75% vs. 95%, P = 0.005) and, when cancer stage, socioeconomic status, comorbidities and cancer treatment were taken into account, they experienced greater risk of death from head and neck cancer (HR 1.88, 1.10, 3.22) and from all other causes (HR 5.83, 95% CI 1.09, 31.04).

**Conclusion:**

These findings show for the first time that Indigenous Australians with head and neck cancer receive less cancer treatment and suggest survival disparity could be reduced if treatment uptake was improved. There is a need for a greater understanding of the reasons for such treatment and survival disparities, including the impact of the poorer overall health on cancer outcomes for Indigenous Australians.

## Background

Overall, Indigenous Australians with cancer are diagnosed with more advanced disease, receive less cancer treatment and have poorer cancer survival than non-Indigenous Australians [1,2]. However, the prognosis for Indigenous people with specific cancers such as those of the head and neck is largely unknown.

The term "cancers of the head and neck" refers to a group of cancers that occur on the lip, tongue, gum, oral cavity, the sinuses, nose, salivary glands and throat [3]. Most head and neck tumours are squamous cell carcinomas (SCC) for which significant risk factors include smoking and alcohol exposure, and increasingly, infection by human papillomavirus (HPV), which now reportedly accounts for 25% of cases in the United States [4]. Head and neck cancers rank as the fifth most commonly occurring cancer type for Australia overall [5] and also for Indigenous people in Queensland [6].

Little is known about the incidence and mortality of head and neck cancer among Indigenous relative to non-Indigenous populations in the developed world. Overall incidence of oral and pharyngeal cancers amongst American Indians/Alaskan Natives (AI/AN) was reportedly lower than for whites but varied widely across districts of the United States, and stage distribution was found to be less favourable for AI/AN [7]. In the Indigenous population of the Northern Territory of Australia, the incidence of head and neck cancer is around 32.78 per 100,000 compared to 19.5 per 100,000 for the rest of Australia [8] and higher incidence and mortality from cancer of the oropharynx have previously been reported in the Northern Territory Indigenous population when compared to non-Indigenous people [2]. Whilst incidence was reportedly similar among Indigenous and non-Indigenous people with head and neck cancers in Queensland between 1997 and 2006, mortality was more than three times greater for Indigenous people [6].

It is not known to what extent established prognostic factors such as late stage at diagnosis, the presence and severity of comorbidities or differences in cancer treatment account for the excess mortality among Indigenous Australians with head and neck cancer. Therefore we conducted this population-based study to compare these factors and survival between Indigenous and non-Indigenous Australians diagnosed with this disease.

## Methods

### Subjects

The study was conducted in Queensland, the north-eastern state of Australia, which has a population of 4.5 million, 3.6% of whom identify as Aboriginal, Torres Strait Islander, or both (respectfully referred to here as Indigenous) [9]. All Indigenous people (over 18 years) residing in Queensland and diagnosed with head and neck cancer between 1998 and 2004 were identified through the population-based Queensland Cancer Registry (QCR). To improve the efficiency of this cohort study, Indigenous patients were matched to a random sample of 74 non-Indigenous people with incident head and neck cancer also identified through the Cancer Registry, of corresponding age (within +/- 5 yrs), sex and location of residence (in terms of degree of remoteness from a major centre).

Clinical data were abstracted from the records at 14 public hospitals to a standard form by one of the researchers (SPM), an experienced nurse, and a former Registrar of the Queensland Cancer Registry. Where records were insufficient, further data were extracted from secondary public hospitals' records. Records were reviewed for diagnostic details such as date, histology and method of diagnosis, cancer stage, treatment and presence of comorbidities. Cancer staging was documented by treating doctors as either Tumour, Nodes and Metastasis (TNM) scores, American Joint Committee on Cancer Staging Scores (I - IV) [10] or as localised cancer, regional spread or metastatic disease; for comparability, TNM and AJCC scores were converted to localised/regional/distant spread using commonly accepted cut- points.

Treatment type, including surgery, radiotherapy and chemotherapy were recorded, as was treatment intention (any intent, curative intent or intention not known). Date, duration and quantity of treatment were also collected. The presence or absence of comorbid conditions was recorded, and a modified Charlson Comorbidity Index Score (referred to here as 'comorbidity score') was assigned (based on severity and number of comorbid conditions and were grouped thus: No score, 1, 2-5 and 5+). Specifically, diabetes, cardio and cerebrovascular disease, hypertension, respiratory illness, renal disease, history of previous cancer and other serious comorbidities that were accepted in the Charslon Comorbidity Scoring framework, were assessed [11]. A Socioeconomic score was assigned using the Socio-Economic Index For Areas (SEIFA) index (based on geographical areas of residence ranked into quintiles: 1 representing the most disadvantaged, and 5 the most advantaged) [12]. Date and cause of death were obtained from the Australian National Death Index.

### Statistical methods

Pearson's Chi-squared analysis or Fisher's Exact test were used for categorical data (proportions), t-test for normally-distributed data (means), and non-parametric tests (Kruskal-Wallis Test) for non-normally-distributed data (medians). Crude and adjusted survival analyses were conducted using Cox proportional hazard models. Hazard ratios were adjusted for stage of cancer at diagnosis, presence and severity of comorbidities, socioeconomic status and 'any' or 'curative' cancer treatment and treatment mode, defined as any curative surgery, radiotherapy or chemotherapy or combination of these. The reference category was "non-Indigenous" unless otherwise stated. All analyses were calculated using Statistics Package for the Social Sciences (PASW) for Windows version 17.0.

Ethical approval to conduct the study was obtained from the Queensland Health Department, all hospitals where data collection took place, and the Queensland Institute of Medical Research. An Indigenous Reference Group was established to inform the study investigators about cultural matters and the translation of results to the community.

## Results

### Demographics and Clinical features

As all Indigenous patients had attended a Queensland public hospital, two non-Indigenous patients who were primarily treated in a private facility were excluded. In addition, cases with cancer of the lip, which mostly arises on the sun-exposed non-mucosal epithelium in white-skinned people [13], were excluded as 7 of the 8 cancers of the lip in the study occurred in the non-Indigenous cohort. Medical records were not available for two Indigenous and three non-Indigenous patients, leaving 67 Indigenous and 62 non-Indigenous people with head and neck cancers for study.

Mean age was 56 years for Indigenous and 55 years for non-Indigenous people and there was no difference in the proportion of males and females, degree of remoteness from a major centre or socioeconomic index between the two groups (Table 1). The percentages of Indigenous and non-Indigenous patients with squamous cell carcinoma were similar (Indigenous 96% vs. non-Indigenous 90%). There was no significant difference in stage at diagnosis (Table 1): the majority of people were diagnosed with regional spread of their cancer (Indigenous 57%, non-Indigenous 65%) and no Indigenous or non-Indigenous people from urban regions were diagnosed with a late stage cancer. There was little difference in the comorbidity score between Indigenous and non-Indigenous people (Table 1) and although a greater proportion of Indigenous people had diabetes (12% vs. 3%), this difference was not statistically significant (P = 0.09).

**Table 1:** Demographic and clinical details, for Indigenous and non-Indigenous patients with cancer of the head and neck in Queensland Australia, 1998- 2004

	Indigenous Status		P value
	Indigenous N = 67 N %	Non-Indigenous N = 62 N %	
**Cancer site (ICD code)**			
Floor of Mouth (C04)	5 (8)	7 (11)	
Gum (C03)	1 (2)	3 (5)	
Hypopharynx (C13)	1 (2)	2 (3)	
Lip, oral cavity, pharynx - ill defined (C14)	9 (13)	6 (10)	
Nasopharynx (C11)	3 (5)	2(3)	
Oropharynx (C10)	5 (8)	2 (3)	0.26
Palate (C05)	8 (12)	2 (3)	
Parotid (C07)	3 (5)	7 (11)	
Pyriform Sinus (C12)	7 (10)	7 (11)	
Tongue (C01- C02)	15 (22)	20 (32)	
Tonsil ( C09)	5 (8)	4 (7)	
Unspecified Salivary glands (C08)	1 (2)	0	
Unspecified mouth (C06)	4 (6)	0	
**Age**			
18- 39	2 (3)	4 (7)	
40-59	40 (60)	39 (63)	0.52
60+	25 (37)	19 (30)	
**Sex**			
Male	47 (70)	43 (70)	0.92
Female	20 (30)	19 (30)	
**Area of remoteness (ARIA)**			
Highly accessible	11 (17)	7 (11)	
Accessible	9 (14)	10 (16)	0.28
Moderately accessible	25 (37)	33 (53)	
Remote	9 (13)	6 (10)	
Very remote	13 (19)	6 (10)	
**Socioeconomic status (SEIFA )**			
1 Most disadvantaged	26 (39)	13 (21)	
2 Low to Intermediate advantage	13 (19)	20 (32)	
3 Intermediate advantage	13 (19)	13 (21)	0.19
4 Advantaged	11 (16)	12 (19)	
5 Most advantaged	4 (6)	2 (3)	
9 Not known	0	2 (3)	
**Stage at diagnosis (SEER score)**			
Localised cancer	23 (34)	15 (24)	
Regional Spread	38 (57)	40 (65)	0.41
Distant metastasis	5 (7)	7 (11)	
Not sure	1 (2)	0	
**Charlson Comorbidity Index (Scores grouped)**			
0	42 (63)	42 (68)	
1	16 (24)	8 (13)	0.30
2- 5	9 (13)	11 (18)	
5+	0	1 (2)	
**Specific comorbidities**			
**Diabetes**			
No	59 (88)	60 (97)	0.09
Yes	8 (12)	2 (3)	
**Hypertension**			
No	55 (82)	47 (76)	0.39
Yes	12 (18)	15 (24)	
Circulatory disease*			
No	61 (91)	51 (82)	0.19
Yes	6 (9)	11 (18)	

*Cardiovascular or cerebrovascular disease

Indigenous people with cancer of the head and neck were significantly less likely to receive any cancer treatment (either palliative treatment, treatment with curative intent or treatment with intention unknown) than their non-Indigenous counterparts (75% vs. 95%, P = 0.005). Furthermore, when people with metastatic cancer or uncertain stage were excluded, Indigenous patients were less likely to receive treatment with curative intent (48% vs. 76%, P = 0.002) (Table 2). Compared to non-Indigenous patients, the pattern of curative treatment received by Indigenous patients was significantly different (P = 0.03). The most common modes of treatment for Indigenous patients were surgery only (36%) or surgery and radiotherapy (25%) compared to surgery and radiotherapy (40%) and chemotherapy (30%) for their non-Indigenous counterparts (Table 2).

**Table 2:** Comparison of cancer treatment given, recurrence, time to death and cause of death, by Indigenous status

	Indigenous N = 67 N (%)	Non-Indigenous N = 62 N (%)	P value
**Any Treatment Given**			
Treatment	50 (75)	59 (95)	
No Treatment	15 (22)	3 (5)	0.005
Not sure	2 (3)	0	
Curative treatment given *	**N = 59**	**N = 53**	
Curative treatment	28 (48)	40 (76)	0.002
No curative treatment	31 (52)	13 (24)	
Mode of curative treatment *			
Surgery only	10(36)	4 (10)	
Chemotherapy only	1 (4)	0	
Radiotherapy only	5 (18)	4 (10)	0.03
Surgery and radiotherapy	7 (25)	16 (40)	
Surgery, chemotherapy and radiotherapy	0	4 (10)	
Chemoradiotherapy	5 (18)	12 (30)	
**Recurrence**	**N = 67**	**N = 62**	
Recurrence recorded	13 (19)	14 (23)	
No recurrence	49 (73)	45 (72)	0.77
Not enough information	5 (8)	3 (5)	
**Time from diagnosis to death**			
Alive at 31^st ^Dec 2006	14 (21)	30 (48)	
Deceased in < 3 months	14 (21)	4 (7)	
Deceased 3 month to 12 months	21 (31)	10 (16)	0.003
Deceased 12 months to 2 years	11 (17)	8 (13)	
Deceased greater than 2 years	7 (10)	10 (16)	
**Cause of death**	**N = 53**	**N = 32**	
Cancer death	44 (83)	29 (91)	0.33
Non cancer death	9 (17)	3 (9)	

*Metastatic cases excluded

The characteristics of those who did not receive treatment were examined more closely. Indigenous people aged over 60 were less likely to receive any treatment than non-Indigenous people of similar age (40% did not receive treatment compared to 5%), as were Indigenous men when compared to non-Indigenous men (30% vs. 5%). Indigenous people with regional spread of disease (27% vs. 5%), those from urban, rural and remote areas and those from all socioeconomic strata, were also less likely to receive any treatment than their non-Indigenous counterparts. The comorbidity score was similar for Indigenous people and non-Indigenous people who did not receive treatment. When only cases with non-metastatic disease were compared, older Indigenous people (42% vs. 31%), those from the most accessible areas (26% vs. 8%), those from the most remote areas (32% vs. 15%), and the most socially disadvantaged (55% vs. 31%) were less likely to receive treatment with curative intent than their non-Indigenous counterparts. 46% of Indigenous people with a comorbidity score of zero did not receive treatment compared to 68% of non-Indigenous people with the same score but the numbers were too small to show significance (data not shown).

The pattern of curative treatment received by Indigenous patients without metastatic disease was significantly different from that of non-Indigenous patients (Table 2). However there were no significant differences in time to surgery or radiotherapy for those who received cancer treatment and Indigenous people were as likely to complete radiotherapy and received a similar course of radiotherapy as their non-Indigenous counterparts.

### Survival

There was no statistically significant difference between the groups regarding recurrence recorded in the medical chart (Table 2). The pattern of death was significantly different when Indigenous patients were compared to non-Indigenous patient; 79% of Indigenous patients were deceased by the end of the follow up period compared to 52% of non-Indigenous patients (Table 2) with risk of all cause-specific death doubled for Indigenous people (HR 2.19 95% CI 1.36, 3.53) (Table 3). When time to cancer death was adjusted for cancer stage, the hazard ratio was 2.45 (95% CI 1.51, 3.96), with little variation when further adjusted for socioeconomic status and comorbidities. When further adjusted for overall treatment, the difference was less but remained statistically significant (HR 1.88% CI 1.10, 3.22). However, when curative treatment and mode of treatment were taken into account, the survival difference was no longer evident (Table 3).

**Table 3:** Proportional hazard ratios, using Cox regression models, of time to death for head and neck cancer patients diagnosed between 1998 and 2004, for Indigenous people in Queensland (reference category is last for all variables)

	HR	(95% CI)
Crude Time to all-cause death	**2.50**	(1.59, 3.93)
Crude Time to cancer-specific death:	**2.19**	(1.36, 3.53)
Adjusted for: Stage^	**2.45**	(1.51, 3.96)
Stage, SEIFA^^	**2.41**	(1.47, 3.93)
Stage, SEIFA, CCI score #	**2.35**	(1.42, 3.78)
Stage, SEIFA, diabetes	**2.34**	(1.42, 3.85)
Stage, SEIFA, CCI score, any treatment	**1.88**	(1.10, 3.22)
Stage, SEIFA, CCI score, curative treatment	**1.59**	(0.92, 2.73)
Stage, SEIFA, CCI score, mode of treatment##	**1.50**	(0.86, 2.64)
Crude Time to non-cancer death	**7.33**	(1.52, 35.1)
Adjusted for: any treatment	**5.83**	(1.09, 31.04)

^ Stage: 1 = localised, 2 = regional spread, 3 = distant metastasis, 4 = not known

^^ SEIFA: 1 = Most disadvantaged, 2 = Mod disadvantage, 3 = Most Advantaged

# Charlson Comorbidity Index (CCI) score: 1 = 0, 2 = 1, 3 = 2-5, 4 = 5+

## Modes of treatment: 0 = No Treatment 1 = surgery, 2 = chemotherapy only, 3 = radiotherapy only, 4 = surgery and radiotherapy, 5 = surgery, chemotherapy and radiotherapy, 6 = chemoradiation

Indigenous patients were also more likely to die of causes other than cancer when compared to non-Indigenous patients (n = 9 (17%) vs. n = 3, (9%)), though the difference was not statistically significant (Table 2). When we compared time to death from other causes between the two groups (Table 3), we found head and neck cancer Indigenous patients to be 7 times more likely than non-Indigenous patients to die of causes other than the cancer after diagnosis (HR, unadjusted, 7.33 95% CI 1.52, 35.18, P = 0.01) and this was largely unaffected when adjusted separately for cancer stage, presence and severity of comorbidities and diabetes (data not shown), or after adjustment for any cancer treatment (fully adjusted HR 5.83 95% CI 1.09, 31.04, P = 0.039).

## Discussion

This study reports clinical characteristics, treatment and survival for 67 Indigenous people diagnosed with head and neck cancer between 1998 and 2004 in Queensland, Australia. As the number of cases was small, and the data collection restricted to information available from medical records, the study is largely descriptive in nature and limited in interpretive power. As a result of matching, Indigenous and non-Indigenous people with head and neck cancer in this study shared a similar demographic profile for location of residence, age and sex, and all attended Queensland public hospitals. We found that only 7% of Indigenous and 11% of non-Indigenous patients were diagnosed with metastatic disease, in keeping with reports that advanced stage at diagnosis is relatively uncommon for head and neck cancer [14].

Overall, Indigenous people with head and neck cancer were less likely to receive any cancer treatment and when only cases with non-metastatic disease were compared, the treatment disparity was even greater. How this compares with other Indigenous populations with head and neck cancer is not known as no similar published data are available. However, treatment bias among Indigenous people with cancer in general, and with other specific cancers, has been reported in a number of studies [1,15-17]. The reasons why Indigenous people in this study were less likely to receive treatment are not precisely known but we found that those Indigenous people who do not receive treatment were likely to be older, male and socially disadvantaged compared to non-Indigenous people who did not receive treatment. Indigenous people were also less likely to receive curative treatment than their non-Indigenous counterparts.

A number of barriers to the use of cancer and other health services for Indigenous Australians have been identified in the literature, and include lack of proximity, availability and cultural appropriateness of health services, lack of specialist care, transport, health insurance and health services affordability, as well as inadequate proficiency in English [16,18-20]. In general these details were not available from the medical records, and are therefore not assessed in this study. Reports suggest factors such as late stage at diagnosis [17], greater prevalence of comorbidities [1] and disparate treatment decisions made by health professionals based on assumptions about socioeconomic and cultural factors [21] may explain some of the treatment differential, but reports are not conclusive.

There was no significant difference in cancer stage or comorbidities between Indigenous and non-Indigenous patients in the present study that might account for the treatment differential, and although we are confident that the medical records were a reliable source of information on cancer stage and the most important comorbidities such as those included in the Charlson index (e.g. diabetes, cardiovascular disease), we acknowledge that our study was small and consequently had limited power to detect small differences between the groups with certainty; consequently some differences may not have been detected. However, it is well known that there is a greater overall burden of comorbidities among the Australian Indigenous population in general and in those with cancer [1,15] and given that Indigenous head and neck cancer patients in this study were 7 times more likely to die from causes other than cancer than non-Indigenous patients, and around 6 times more likely to die from other causes after accounting for any cancer treatment received, it is plausible that chronic ill-health is associated with less treatment.

We acknowledge some possible under-representation of Indigenous patients in this study due to misclassification of Indigenous status at the QCR; however a data quality audit of Queensland hospitals, who provide cancer notification to the QCR, suggest a high level of accurate identification, so this is likely to be minimal [22]. We also believe the Indigenous to non-Indigenous comparison to be internally valid, with little, if any, misclassification of Indigenous status in the study sample, as medical charts were carefully reviewed to verify Indigenous status and discrepant cases were excluded. Unfortunately smoking history was not routinely available from the medical records for this study and we were therefore unable to investigate possible associations between smoking and head and neck cancer for this population. As up to 40% of Indigenous people are reported to smoke and smoking has been strongly associated with head and neck cancer [23], we recommend more thorough collection of smoking data in the health records of Indigenous patients to enable better assessment of the long-term impact on the health of this population.

Indigenous people who did receive treatment were significantly less likely to receive surgery with adjuvant radiotherapy, the most commonly administered mode of treatment for head and neck cancer in the early stages [4]. However, as mode of treatment varies depending on the tumour site and extent and, as the distribution of cancer types differed somewhat among Indigenous and non-Indigenous people in the present study, it was difficult to compare treatment modes between the two groups. When those who had treatment were compared, there was no difference in dose, duration or completion rate of radiotherapy between Indigenous and non-Indigenous people with head and neck cancer and overall, time to any treatment and curative treatment was also similar. Although numbers were small, the implication is that once engaged in treatment, the treatment pathway was likely to be similar between Indigenous and non-Indigenous people in the public setting in Queensland. This accords with a 2006 report by Condon and colleagues who found that whilst Indigenous patients were less likely to be recommended treatment, there was no significant difference in completion [15].

When cancer-specific death was adjusted for socioeconomic status, the presence and severity of comorbidities and cancer stage, the survival difference remained the same and changed little when adjusted for treatment. However, when curative treatment and mode of treatment were taken into account, the survival difference was no longer significant. This might suggest that if the same treatment was offered, Indigenous people would have similar survival from head and neck cancer as non-Indigenous people. Although the numbers in our cohort were small and so the results lacked statistical precision, we identified disparities in cancer treatment delivery which should be further investigated and addressed by health service personnel. Although survival from head and neck cancer in Indigenous populations has not been reported elsewhere, the results of this study are consistent with the poor cancer survival and worse mortality that has been extensively reported for Indigenous people in Australian with other cancers [24,25]. We were unable to adjust for the presence of HPV which has been found to be a predictor of survival in whites in America [26] as HPV testing was not routinely undertaken at the time of the study.

## Conclusions

Despite the small sample size, the study obtained statistically significant and clinically important differences in treatment and survival in Indigenous people with head and neck cancer in Queensland Australia. Indigenous people treated in the public health system in Queensland were similar in socioeconomic status and stage of disease to non-Indigenous people and received less treatment and had poorer survival than non-Indigenous people with head and neck cancer. Further study is needed to ascertain to what extent the observed differences may be explained by differences in the true burden of comorbidities in Indigenous and non-Indigenous people.

## Abbreviations

AJCC: American Joint Committee on Cancer; HPV: Human papillomavirus; HR: Hazard ratio; PASW: Statistics Package for the Social Sciences; SCC: Squamous Cell Carcinoma; SEIFA: Socio-economic Indexes for Area; TNM: Tumour, nodes, metastasis; US: United States.

## Competing interests

The authors declare that they have no competing interests.

## Authors' contributions

SM made contributions to conception and design, substantial contribution to the acquisition of data, conducted the analysis, interpreted the data and drafted the manuscript. PV made substantial contributions to conception and design, reviewed the analysis and contributed to the interpretation of the data. AG made contributions to conception and design, contributed to the interpretation of the data. GG made contributions to the interpretation of the data. MC made contributions to conception and design, contributed to the interpretation of the data. All were involved in revising it critically for important intellectual content and have given final approval for this version to be published.

## Pre-publication history

The pre-publication history for this paper can be accessed here:

http://www.biomedcentral.com/1471-2407/11/460/prepub
